# Evolving Concepts of the SCORE System: Subtracting Cholesterol from Risk Estimation: A Way for a Healthy Longevity?

**DOI:** 10.3390/life14060679

**Published:** 2024-05-24

**Authors:** Francesco Natale, Rosa Franzese, Luigi Marotta, Noemi Mollo, Achille Solimene, Ettore Luisi, Carmine Gentile, Francesco S. Loffredo, Paolo Golino, Giovanni Cimmino

**Affiliations:** 1 Vanvitelli Cardiology Unit, Monaldi Hospital, 80131 Naples, Italy; natale.francesco@ospedalideicolli.it (F.N.); rosa-franzese@libero.it (R.F.); luigi.marotta@studenti.unicampania.it (L.M.); noemi.mollo@studenti.unicampania.it (N.M.); achille.solimene@unicampania.it (A.S.); ettore.luisi@studenti.unicampania.it (E.L.); carmine.gentile@studenti.unicampania.it (C.G.); francesco.loffredo@unicampania.it (F.S.L.); paolo.golino@unicampania.it (P.G.); 2 Department of Translational Medical Sciences, Section of Cardiology, University of Campania Luigi Vanvitelli, 80131 Naples, Italy; 3 Cardiology Unit, AOU Luigi Vanvitelli, 80138 Naples, Italy

**Keywords:** low-density lipoprotein cholesterol, atherosclerosis, causal risk factor, atherosclerotic cardiovascular diseases

## Abstract

The role of cholesterol, mainly low-density lipoproteins (LDL-C), as a causal risk factor for atherosclerotic cardiovascular disease (ASCVD) is now established and accepted by the international scientific community. Based on this evidence, the European and American guidelines recommend early risk stratification and “rapid” achievement of the suggested target according to the risk estimation to reduce the number of major cardiovascular events. Prolonged exposure over the years to high levels of LDL-C is one of the determining factors in the development and progression of atherosclerotic plaque, on which the action of conventional risk factors (cigarette smoking, excess weight, sedentary lifestyle, arterial hypertension, diabetes mellitus) as well as non-conventional risk factors (gut microbiota, hyperuricemia, inflammation), alone or in combination, favors the destabilization of the atherosclerotic lesion with rupture/fissuration/ulceration and consequent formation of intravascular thrombosis, which leads to the acute clinical manifestations of acute coronary syndromes. In the current clinical practice, there is a growing number of cases that, although extremely common, are emblematic of the concept of long-term exposure to the risk factor (LDL hypercholesterolemia), which, not adequately controlled and in combination with other risk factors, has favored the onset of major cardiovascular events. The triple concept of “go lower, start earlier and keep longer!” should be applied in current clinical practice at any level of prevention. In the present manuscript, we will review the current evidence and documents supporting the causal role of LDL-C in determining ASCVD and whether it is time to remove it from any score.

## 1. Introduction

It is now well established and universally accepted that a high level of cholesterol, mainly the fraction carried by low-density lipoproteins (LDL-C), is the major determinant of atherosclerotic plaque development and progression leading to cardiovascular diseases (CVD) [1,2,3,4]. This association has been reported in experimental studies, epidemiological cohorts, randomized clinical trials on lipid-lowering drugs, and Mendelian randomization studies [5]. Of interest, the correlation between LDL-C levels and the outcomes of atherosclerotic CVD (ASCVD) becomes much closer with the extension of follow-up (in epidemiological studies) [6], the intensity of treatment (in intervention) [7], and its early initiation (in long-term studies) [8,9]. Starting from this evidence, it can be concluded that an individual’s risk of ASCVD is strongly related to their cumulative lifetime exposure to LDL-C [10]. Additional conventional and non-conventional risk factors reinforce the LDL-C role in ASCVD development [11,12]. A significant increased long-term risk of coronary heart disease (CHD) and cardiovascular mortality has also been reported in young adults with LDL-C ≥ 100 mg/dL (2.5 mmol/L) [13]. Based on these considerations, to prevent atherosclerosis and its consequences (myocardial infarction, ischemic stroke, and peripheral arterial disease), it is necessary to act early in life. Indeed, the early manifestations of atherosclerosis are often evident in the third decade of life [14]. This concept is further reinforced by taking into account the morbidity and early mortality associated with familial hypercholesterolemia (FH) [15,16,17,18]. Furthermore, changes in plasma cholesterol levels have been found to be directly associated with CVD events in young adults [19]. Based on these results and the evidence from lipid-lowering trials [7,20,21,22,23], it is clear that, regarding LDL-C, “lower is better, earlier and for longer”. In the present article, we will discuss the main recommendations for the management of LDL-C levels in light of the targets to achieve according to the risk estimation in light of the current guidelines, with particular attention to the temporal changes in stratification, the variation between primary and secondary prevention, and the need to intensify therapeutic strategies earlier in order to maintain the defined target in the long term. This evaluation will be performed in light of the accepted role of LDL-C as a causal factor in ASCVD.

## 2. Literature Sources and Search Strategy

We performed a non-systematic review of the literature by applying the search strategy to different electronic databases (MEDLINE, EMBASE, Cochrane Register of Controlled Trials, and Web of Science). Original reports, meta-analyses, and review articles in peer-reviewed journals up to April 2024 addressing the role of LDL-C in primary and secondary prevention. LDL-C, atherosclerosis, cardiovascular risk, primary and secondary prevention, and lipid-lowering interventions were incorporated into the electronic databases for the search strategy. The references of all identified articles were reviewed to look for additional papers of interest to extrapolate the more recent available data on the link between LDL-C and cardiovascular risk.

## 3. LDL-C in Primary Prevention: The Goal for Long-Term Event-Free Survival

According to the most recent European and American guidelines, the indication for the treatment of dyslipidemia, as well as other cardiovascular (CV) risk factors, in primary prevention depends on the risk of CVD and the risk/benefit ratio expected from treatment over a lifetime; in fact, the greater the risk of disease, the greater the benefit that can be obtained by starting treatment earlier [24,25]. Hence, CV risk estimation is the keystone for decision-making in primary prevention. The guidelines recommend its application in men over 40 and women over 50 without known risk factors for ASCVD, as well as in all patients with known major CV risk factors [25]. It is also important to point out that a CVD risk still remains over time even when all risk factors are at goal [26,27,28,29].

### 3.1. Cardiovascular Risk Estimation: Is It Limited to a SCORE Definition?

For the general purpose of risk stratification, current guidelines recommend the use of specific scores to estimate the risk of fatal and non-fatal CV events at 10 years classify patients into CV risk categories using cut-off values that are different for each age group [25]. In particular, the European guidelines recommend the use of the Systematic Coronary Risk Evaluation SCORE2 in apparently healthy patients (without established CVD or diabetes or other major comorbidities) from 40 to 69 years of age, replaced by the SCORE2-OP in patients aged 70 years or older [25], while the American guidelines, for the same purpose, recommend the PCE (Pooled Cohort Equation) score for patients aged 40 to 75 years [30]. These scores allow us to categorize patients as having moderate-low, high, or very high CV risk. Patients who have major comorbidities, such as familial hypercholesterolemia (FH) or at least moderate chronic renal impairment, must be considered directly at high or very high CV risk [24,25]. Patients with type 2 diabetes mellitus (T2DM), according to the latest European guidelines, start from a moderate CV risk that further increases according to the time of diabetes, target organ damage (defined as EGFR < 45 mL/min or EGFR between 45 and 59 mL/min in association with microalbuminuria or 24-h proteinuria >300 mg/g or the presence of microvascular damage in at least three different sites, e.g., microalbuminuria, retinopathy, and neuropathy), documented ASCVD, and the application of SCORE 2-Diabetes, which integrates information on conventional CV risk factors with diabetes-specific information [31].

The final LDL targets to be achieved are different for each risk class: in those at moderate-low risk, an LDL < 100 mg/dL is desirable; in those at high risk, LDL should be <70 mg/dL with a reduction of at least 50% from baseline; in those at very high risk, LDL should be <55 mg/dL with a reduction of at least 50% from baseline [25].

In addition to the scores recommended by the guidelines, there are also other cardiovascular risk calculators such as the Quality and Outcomes Framework (QOF) Risk 3 (QRISK3) which is the latest update of a model to calculate the risk of developing cardiovascular diseases such as stroke or heart attack at 10 years, based on an algorithm validated on a large UK population aged 25–84, and which takes into account a number of factors already included in QRISK2 including age, gender, high blood pressure, diabetes, hypercholesterolemia, smoking, family history of cardiovascular disease, and new factors including stage III to IV chronic renal failure, corticosteroid use, diagnosis of systemic lupus erythematosus, severe mental illness, treatment with atypical antipsychotics [32]. Based on this algorithm, the percentage of risk of developing a stroke or heart attack in the next 10 years is obtained (https://www.qrisk.org/index.php, accessed on 17 May 2024). A score > 10% indicates that the initiation of lipid-lowering therapy should be considered in primary prevention (www.nice.org.uk/guidance/ng238, accessed on 17 May 2024). The QRISK is the predictor of cardiovascular risk currently recommended in the UK by the National Institute for Health and Clinical Excellence (NICE) as opposed to the NICE version of the Framingham risk score [33], but although it has been shown to be very adequate in discriminating the general population, it has been shown to perform poorly in older patients and patients with multiple comorbidities, in whom it would overestimate cardiovascular risk by not taking into account concurrent mortality risk [34].

According to the guidelines, treatment in primary prevention of dyslipidemia, as a cardiovascular risk factor, is recommended in apparently healthy “very high CV risk” patients who have SCORE2 greater than or equal to 7.5% (age group < 50 years) or greater than or equal to 10% (age group 50–69 years) or SCORE2-OP greater than or equal to 15% (age group > 70 years), while it should be considered in apparently healthy “high CV risk” patients who have SCORE2 between 2% and 7.5% (age group < 50 years) or between 5% and 10% (age group 50–69 years) and may be considered in those with SCORE2-OP between 7.5% and 15% (age group > 70 years), with regard to assessment of CV modifiers and lifetime risk/benefit evaluation [25]. In subjects at moderate-low risk (SCORE 2 < 2.5% if age < 50 years or <5% if age 50–69 years), treatment is generally not indicated, unless they have major risk modifiers or an absolute lifetime benefit is demonstrated [25].

**Figure 1:**
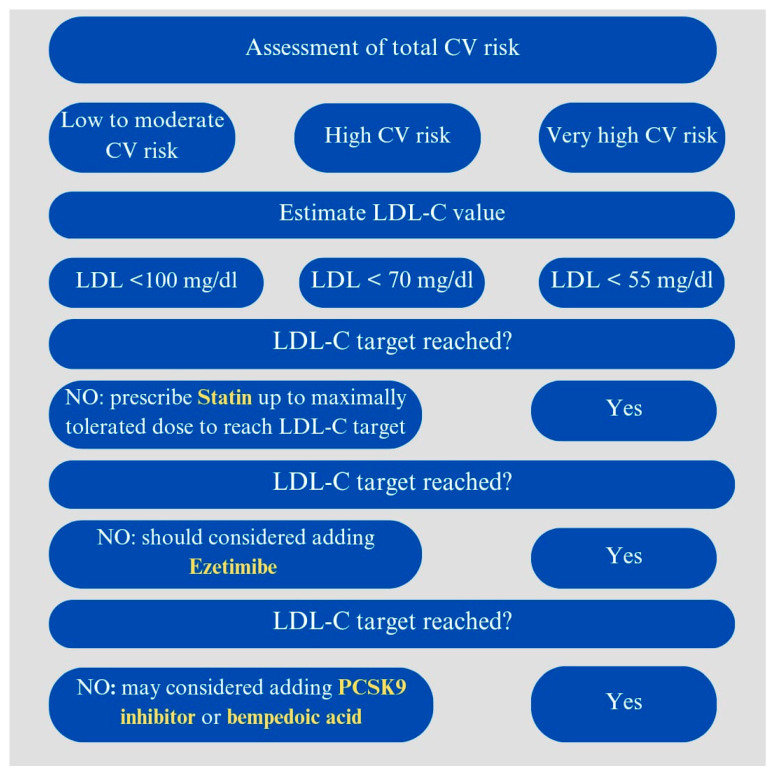
Lipid-lowering strategies and targets to achieve based on CV risk assessment. In yellow the medication suggested at each step.

A schematic view of the stepwise approach suggested by the current guidelines is shown in Figure 1.

In diabetic patients, the guidelines recommend achieving the targets according to CV risk stratification, indicating statins as the first line of choice in patients who have LDL-C values above the targets [25,31].

European guidelines [25,35] recommend as a first step to exclude potential secondary causes of dyslipidemia, particularly hypothyroidism, alcohol abuse, Cushing’s syndrome, liver or kidney diseases, and the use of certain drugs such as corticosteroids. They also suggest modifying lifestyle, increasing physical activity, and changing diet (increasing consumption of fruits, vegetables, nuts, fish, whole grains, and reducing red meats and refined carbohydrates) [25,35]. They further suggest, in primary prevention, prescribing high-intensity statin therapy to achieve the LDL-C target based on the specific risk category and, in cases of failure or statin intolerance, considering the addition of ezetimibe [25,35]. Furthermore, in patients at very high risk who do not reach the LDL-C target with maximum statin and ezetimibe therapy, PCSK9 inhibitors may be considered [25,35].

### 3.2. Lipid-Lowering Strategy: The Way to the Target

Alongside traditional lipid-lowering therapies (LLT), several new drugs have been approved by the FDA for the treatment of dyslipidemias, and a growing number of studies are underway to evaluate their efficacy and safety profiles. Bempedoic acid, the first inhibitor of the ATP citrate lyase class, represents a new lipid-lowering drug that fits into our therapeutic armamentarium [36]. The CLEAR Outcomes study [37] was the first to evaluate the efficacy of bempedoic acid in primary prevention in statin-intolerant patients. In a subpopulation of the study (each study group included about 30% of patients with high and very high cardiovascular risk for primary prevention), it showed a significant reduction in the primary composite endpoint (HR 0.68, 95%CI: 0.53–0.87); however, it is necessary to consider that the number of events observed in these patients was low (n: 111 for bempedoic acid), and therefore these results are not conclusive. However, the international lipid expert panel in a position paper concludes that although there are not yet enough data available on the role of bempedoic acid in primary prevention, its favorable effects on glycemia and inflammatory markers make this drug a rational choice in the care of specific patients in primary prevention [38].

On the other hand, the randomized, placebo-controlled ORION-11 study [39] evaluated the efficacy of inclisiran, a small interfering ribonucleic acid (siRNA) therapy targeting PCSK9 production, in a population of high-risk patients in primary prevention whose LDL levels were high despite the use of the maximum tolerated doses of statins. At day 510, the placebo-corrected LDL-C change with inclisiran was −43.7% (95% confidence interval), with a corresponding time-adjusted change of −41% (95% CI; *p* < 0.0001). Therefore, inclisiran has proven to be a well-tolerated drug in patients in primary prevention and has determined a significant reduction in atherogenic lipoprotein levels with the maintenance dose twice a year [39].

### 3.3. Low-Density Lipoprotein Cholesterol: Standing Alone or Better Together?

Current guidelines assimilate the treatment of dyslipidemia in primary prevention to any other CV risk factor [24,25,35]. However, plenty of evidence over the last two decades has largely demonstrated the causal role that LDL-C plays in the development and evolution of ASCVD [5,40]. It has been reported that LDL-C levels between 25 and 40 mg/dL, which are only typical at birth [41] or in hunter-gatherer populations [42], are associated with a low risk of developing atherosclerosis. The risk of ASCVD is closely related to cumulative LDL-C-dependent exposure, i.e., the magnitude of elevated LDL-C multiplied by years of exposure [43]. This evidence is also supported by Mendelian studies that have shown that long-term exposure to congenitally lower levels of LDL-C is associated with a lower CV risk than the reduction achievable with drug therapy, which is generally started too late, when subjects already have atherosclerotic plaques formed because they have had a high cumulative exposure to LDL-C [44]. The minimum LDL-C target to be reached in order to prevent the progression of atherosclerotic disease and its clinical manifestation is still unclear, but it has been shown that reducing LDL-C leads to a reduction in CV risk; in particular, a reduction in each 1 mmol/L (38.7 mg/dL) of LDL-C leads to a 22% reduction in the risk of CV events [45]. Intravascular diagnostic studies have shown that atherosclerotic plaque progression stops when LDL-C levels are <70 mg/dL [43]. The same kind of studies in patients treated with PCSK9 inhibitors (PCSK9i) have shown plaque progression in patients with LDL-C levels between 20 and 30 mg/dL [46]. According to the available data, primary prevention interventions do not seem to be sufficient to control the onset of atherosclerotic disease. According to the American Heart Association’s Annual Update of Heart Disease and Stroke, the prevalence of CVD (CV disorders including myocardial infarction, heart failure, and stroke) between 2017 and 2020 among adults was 48.6% of the population, and during the same period, among adults, 32.8% of males and 36.2% of females had total cholesterol greater than or equal to 200 mg/dL and 25.6% of males and 25.4% of females had LDL-C greater than or equal to 130 mg/dL [47].

It is important to point out that LDL-C is a heterogeneous mixture of particles differing in structure, density, size, and atherogenic properties [48]. Of these, small, dense LDL (sdLDL) is the most atherogenic because of greater arterial entry and retention, higher susceptibility to oxidation, and reduced affinity for the LDL receptor (LDLR) [49]. Current evidence supports the role of sdLDL as an independent risk factor for CVDs, which emphasizes the clinical importance of both the quality and quantity of LDL-C [49]. Other metabolic risk factors are currently being considered in defining LDL-C-related risk, such as Apolipoprotein B (apo-B), lipoprotein (a) (Lp(a)) and LDL-C/HDL-C ratio (LHR). apo-B is a non-exchangeable apolipoprotein exclusively associated with plasma lipoproteins [50]. It is the key structural component of all the atherogenic lipoproteins, including sdLDL [50]. It has been implicated in the development of atherosclerosis, and at the same time, it is also essential for the binding of LDL-C to the LDLR for cellular uptake and degradation of LDL-C [51]. It has been reported that elevated plasma levels of apo-B are directly related to the development of CAD [52]. Furthermore, Lp(a) is a subtype of lipoprotein involved in the transport of cholesterol in the blood [53]. It consists of LDL conjugated to an apolipoprotein(a) [53]. High concentrations of Lp(a) are a risk factor for CV events [53]. Finally, LHR has been recently suggested as an additional indicator of atherosclerotic disease [54], since a high LHR may be a possible risk factor for CHD [54].

The current management of dyslipidemia in primary prevention has therefore weaknesses. The risk classification on the basis of scores has several limitations. First, age, since the management of patients younger than 40 years of age, who are the ones at whom primary prevention should be aimed and who would have a higher cumulative exposure to LDL-C, is not standardized and the use of scores in this age group is not indicated, nor is treatment unless they have major comorbidities such as CKD or FH [24,25]. Second, ethnicity: the scores are calibrated on four groups of countries classified as low, moderate, high, and very high risk in relation to the CV mortality rates studied by the WHO, but many countries are left out of these classifications, particularly those in the Middle East and all of Southeast Asia, as well as ethnic Africans [24,25]. Third, the scores make an assessment based on conventional CV risk factors but do not take into account a whole series of factors that, despite being associated with CV risk, are not included among the conventional ones [12]. In borderline decision-making cases, the guidelines recommend assessing some of these factors as risk modifiers (inflammation, psychosocial stress and mental disorders, family history of CVD, socioeconomic level, air pollution, subclinical presentation of atherosclerosis, and biomarkers associated with an increased risk of ASCVD), but their use is not well standardized [55]. The use of subclinical evidence of atherosclerotic disease is a strategy recommended to personalize treatment choice in patients at moderate or low risk. Among the different possible evaluations (Ankle Brachial Index, Intimal Media Thickness, and carotid plaque assessment), the coronary artery calcium (CAC) assessment at cardiac gated computed tomographic (CT) scanners is the one with the strongest evidence [55,56]. Specifically, a CAC ≥ 100 is an indicator of an increased risk of cardiovascular events, a CAC > 300 is similar to that of patients who already have established ASCVD [57], a CAC of 0, unless found in patients with a strong family history or diabetic or heavy smokers, may induce a delay in LLT [56]. Unfortunately, the application of this method for screening is expensive and difficult to implement on a large scale in clinical practice.

Another putative tool for CV risk estimation might be the evaluation of a genetic profile linked to CAD. It has been reported that nine genetic variants are associated with reduced LDL-C plasma levels [5]. Individuals carrying these variants may get up to a 54.5% reduction in cardiac risk for each 1 mmol/L (38.7 mg/dL) reduction in LDL-C, thus demonstrating that prolonged exposure to low LDL-C levels early in life is associated with a reduction in the risk of CAD compared to the usual practice of lowering LDL-C in a more advanced stage of life [5]. A practical evidence-based approach might be the evaluation of genetic risk scores in all males in their 20s and all females in their 30s and 40s in order to identify individuals at high genetic risk where to implement guidelines recommendations [58], starting with lifestyle modifications [59] ensuring earlier and more effective primary prevention measures.

### 3.4. The Other Side of the Moon: Reducing LDL-C for Social and Economic Impact

The social and economic burden of CVD is significantly increased in all industrialized countries [60,61,62,63,64]. Data on morbidity, mortality, health, social, and informal care indicate that in the European Union, the cost of CVD is estimated to be € 282 billion per year [61]. In the United States, national expenditures for ASCVD are projected to increase by over 2.5-fold by 2035 [64] defining true financial toxicity. Taking into account these analysis, prevention is also essential to guarantee economic sustainability for future generations. In this regard, an interesting report was published early in 2016 addressing the cardiovascular benefits (as the number of MACEs averted) and economic impact (billions of dollars saved) of achieving the goal of LDL-C ≤ 100 mg/dL or ≤70 mg/dL in a 20-year period (from 2015 to 2035) in patients at high CV risk [65]. Keeping LDL-C at the goal of ≤70 mg/dL for the period indicated will result in 14,200 cumulative numbers of MACEs averted $5.1 trillion saved for averted deaths and MACEs vs. 7500 cumulative numbers of MACEs averted and $3.4 trillion saved for averted deaths and MACEs for LDL-C ≤ 100 mg/dL [65]. This analysis further supports the concept of lower LDL-C even in primary prevention to reduce MACEs and the socio-economic burden of ASCVD.

## 4. LDL-C in Secondary Prevention: If the Lower, the Better

The current ESC guidelines on dyslipidemia establish that in patients at very high risk (documented CVD, diabetes mellitus, severe CKD, FH, and a SCORE > 10%), the percentage of LDL-C reduction must be greater than 50% compared to baseline with a suggested target <55 mg/dL (class I level of evidence A) [35]. In addition to the previous 2016 guidelines [66], it was established that in patients who had a second CV event within 2 years despite LLT with high-intensity statins at maximum dosage (atorvastatin 40–80 mg per day or rosuvastatin 20–40 mg per day), the LDL-C level to be achieved should be <40 mg/dL (class IIb level of evidence B) [35], further supporting the role of LDL-C as a true causal factor of CVD. However, data from observational studies reveal that these ambitious goals are not easy to achieve [67]. This gap has multiple explanations. First, the monotherapy is not powerful enough, and recent observational studies clearly indicate that there are still too many patients in monotherapy failing to achieve the recommended target [67,68]. The new therapeutic options currently available aim to improve therapeutic adherence and reach target LDL-C levels more easily. Among these, in addition to ezetimibe and/or bempedoic acid, which are able to reduce LDL-C levels by 18% [69] and 24% [36], respectively, an important role is now played by PCSK 9 inhibition through monoclonal antibodies (alirocumab and evolocumab) [70] and siRNA (inclisiran) [39,71]. Blocking the PCSK9 protein results in increased recirculation of the LDL-C receptor on the cell surface, thus improving its function [70]. Three major clinical programs have evaluated the efficacy and safety of PCSK9i in different CV scenarios: FOURIER, ODYSSEY, and ORION [72]. Specifically, FOURIER, and ODYSSEY trials have analyzed evolocumab and alirocumab, respectively, reporting an average LDL-C reduction by more than 50% in an efficacy and safety manner [73]. Long-term efficacy in LDL-C reduction and safety administration has also been shown for Inclisiran [74]. They are indicated in very high-risk patients in addition to statin therapy at the maximum tolerated dose or as an alternative in cases of intolerance.

### 4.1. The Dream Comes True: Is Zero the Best?

**Figure 2:**
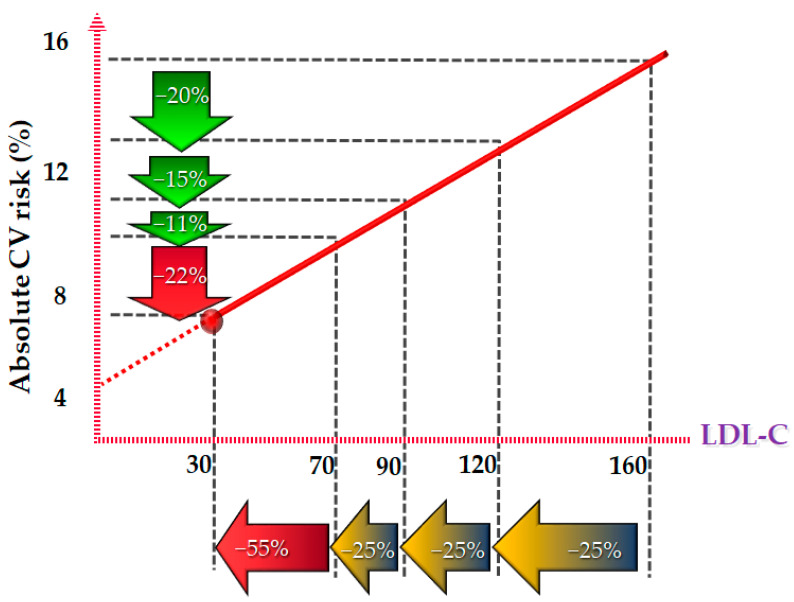
Linear correlation between percentage of LDL-C lowering and percentage of CV benefits.

All the recent clinical studies on LLT support the safety of very low levels of LDL-C. In particular, the use of PCSK9 inhibitors in combination with high-intensity statins has led to an unprecedented reduction in LDL-C, with median LDL levels of 30 mg/dL in the FOURIER study [75,76] and 40 mg/dL within 4 months in the ODYSSEY-OUTCOMES trial [77]. The safety results were reassuring regarding neurocognitive disorders, hemorrhagic stroke, worsening of blood glucose, and/or onset of diabetes mellitus [76,78,79]. In particular, a secondary analysis of the FOURIER study [76] demonstrated a linear relationship between the lowering of LDL cholesterol and the reduction in the primary clinical outcome as well as the secondary outcome, in the absence of anyerious adverse events or any of the other nine prespecified safety events [76]. These results were confirmed in a safety analysis of the FOURIER-OLE study with >8 years of follow-up [78]. A second analysis specifically focused on diabetes [79] showed that evolocumab did not increase the risk of new onset diabetes or worse blood glucose. Already after 12 weeks, 63.2% of patients had achieved an LDL-C level < 40 mg/dL, with a median LDL-C level of 30 mg/dL [9]. The results of the trials on PCSK9i have therefore confirmed that their addition to the standard LLT allows a rapid and significant lowering of LDL-C values, reducing the risk of coronary events and thus confirming the now widely spread concept of “the lower the better”. However, physiologically, cholesterol is a fundamental component of the cell membrane and a precursor of steroid hormones. For this reason, doubts have been raised about any adverse effects linked to exceeding the threshold of 30 mg/dL of LDL-C, and the question has therefore been asked to what level it is actually possible to lower LDL-C without consequences. Patients with extremely low LDL-C levels caused by six genetic conditions were studied, including PCSK9 loss of function and abetalipoproteinemia [80]. Overall, no comorbidities associated with low LDL-C levels have been highlighted. Indeed, it was seen that patients in particular with PCSK9 loss of function had a lower risk of coronary heart disease and, in general, of CV events. It has been hypothesized that low levels of LDL-C would lead to atheroprotection or even regression of the atherosclerotic plaque. A warning about a possible link between LDL-C < 30 mg/dL and an increased risk of hemorrhagic stroke was raised [81], but several other observations have excluded this association [82]. Taking together all the published studies on very low levels of LDL-C, “the zero LDL Hypothesis” is born [83]. It is based on the basic concept that if the main function of LDL metabolism is the excretion of cholesterol, the use of drugs like PCSK9i that lower its blood levels is simply enhancing the LDL-LDLR pathway function. However, data regarding the long-term safety of achieving and maintaining LDL-C levels < 15 mg/dL are still limited. Further studies are therefore needed to confirm the real efficacy and safety of such low LDL-C levels. A schematic view of the CV benefits associated with LDL-C reduction is provided in Figure 2.

### 4.2. Time and Tide Wait for None: The Benefits from Earlier and for Longer

In the FOURIER trial, which enrolled 27,564 patients with ASCVD and LDL-C 70 mg/dL despite optimal LLT, evolocumab significantly reduced LDL-C levels and the risk of MACE over a median follow-up of 2.2 years [75]. However, no benefits were observed in terms of CV or overall mortality within this relatively short follow-up period. Despite this, the risk of myocardial infarction and stroke was notably reduced [75].

In the subsequent open-label follow-up study, FOURIER-OLE, conducted over a median of five years, patients originally assigned to the placebo group, despite the achievement of the same LDL-C levels as the evolocumab arm within 12 weeks of drug administration, experienced fewer benefits over time. Specifically, the original evolocumab group, respected by the later initiator, showed a 15% lower risk of CV death, myocardial infarction, stroke, hospitalization for unstable angina or coronary revascularization, a 20% lower risk of CV death, myocardial infarction, or stroke, and a 23% lower risk of CV death alone [9], indicating that a late achievement of the LDL-C target generates a gap that seems difficult to overcome.

## 5. Familial Hypercholesterolemia: When and How to Manage the LDL-C Levels?

Familial hypercholesterolemia (FH) is an autosomal dominant disease characterized by high levels of plasma LDL-C. Two forms of FH can be described: heterozygous familial hypercholesterolemia (HeFH) and homozygous familial hypercholesterolemia (HoFH). HeFH is the more common one, with a prevalence of around 1/250–300 [84]. FH is caused by loss-of-function mutations in the LDLR gene (around 85–90% of cases) and the apoB gene, or gain-of-function mutations in the PCSK9 gene. An exception as regards the inheritance model of the disease is represented by mutations in the LDLRAP1 gene that cause a recessive phenotype of FH. All these mutations are responsible for the elevation of circulating LDL-C in childhood and adolescence due to a decreased cellular uptake of LDL-C and consequently for the development of early CAD (before the ages of 55 for men and 60 for women). Polygenic mutations and epigenetic mechanisms also play an important role in defining the cause of the disease when unknown mutations are identified. Plasma levels of lipoprotein(a) [Lp(a)] are often increased in FH, especially in HoFH, while high-density lipoprotein-C (HDL-C) is often reduced [84,85]. The diagnosis of FH is clinically made, as defined in the criteria from the Dutch Lipid Clinic Network. However, it can be confirmed using genetic testing. Once an index case is detected, cascade screening can be used to identify new cases. While HoFH is less common than HeFH (its prevalence is around 1/250.000–360.000), it is also a more serious disease because of the exposure to more elevated cholesterol levels (LDL-C levels > 400 mg/dL in untreated patients [86]) from birth and thus earlier developing of ASCVD and aortic stenosis with ostial involvement (around 20 years of age) and its lower survival (around 30 years of age). The aggressive nature and the worse prognosis of this disease demonstrate that absolute LDL-C levels affect the severity of the CV phenotype [85,86,87].

Patients affected by FH should start treatment as early as possible due to the cumulative cholesterol burden. High-intensity statin therapy is the core treatment for the disease, in most cases in combination with ezetimibe. According to 2019 ESC/EAS guidelines, in FH patients at very high risk of ASCVD due to a prior history of ASCVD or another major risk factor, LDL-C goals are a ≥ 50% reduction in LDL-C from baseline and an LDL-C < 55 mg/dL. In the absence of previous ASCVD or another major risk factor, patients with FH are categorized as high-risk, and LDL-C goals are a ≥50% reduction in LDL-C from baseline and an LDL-C < 70 mg/dL [35]. Very-high-risk patients with FH could benefit from PCSK9i if statins plus ezetimibe are not enough to achieve the goal or for statin intolerance [35].

HoFH patients need more intense treatment due to their more severe condition, starting with a combination of high-intensity statin and ezetimibe and including lipoprotein apheresis when medical treatment is not enough. According to the European Atherosclerosis Society’s (EAS) consensus panel, maximally tolerated pharmacological therapy must be maintained [86]. In HoFH, pharmacologic treatment should start as early as possible, at diagnosis, in order to reduce LDL-C levels as much as possible, and thus preventing or delaying the occurrence of CV events and increasing survival. In the most complex cases, liver transplantation is considered an option [86]. In the future, liver-directed gene transfer and gene editing will represent a promising opportunity for patients with HoFH [86]. It is well established that in FH patients, elevated LDL-C levels and the duration of exposure play a key role in the progression of atherosclerosis and ASCVD [88]. Thus, early and intense reduction is mandatory to achieve regression of atherosclerosis and, consequently, a reduction in coronary events and mortality [86]. LLT should be initiated in children [89]. According to ESC/EAS guidelines, at diagnosis, the first step of FH treatment in children is a healthy diet and lifestyle, followed by statins from the age of 6 to 10 years, starting with increasing doses (low to high) in order to reach the targets. The goal in children >10 years of age is an LDL-C < 135 mg/dL, and at younger ages, a ≥50% reduction in LDL-C [86,89]. A very high LDL-C or additional CV risk factors may decrease this target or the age recommended for starting statin therapy [86,89]. According to the 2023 Updated EAS consensus statement, in HoFH children and/or adolescents, the LDL-C goal is <115 mg/dL if treatment is initiated before 18 years, in the absence of ASCVD at imaging assessment, with a lower goal in those with established ASCVD [86]. A summary of the recommendations is reported in Table 1.

Few discrepancies exist between the international guidelines on the age of starting therapy for FH patients. According to the 2014 EAS consensus panel, treatment should be started at 8–10 years of age in children with HeFH [90] and at diagnosis in those with HoFH [86]. The American College of Cardiology–American Heart Association guidelines for familial hypercholesterolemia recommend initiation of statins as young as 10 years [91]. A comparison between guidelines is shown in Table 2.

None of the guidelines recommended the beginning of statins before the age of eight years in HeFH [92]. When recommended, statins, beyond LDL-C reduction, improve endothelial function and reduce the progression of intima–media thickening (IMT) [92,93,94]. Many concerns were expressed about statin safety in very young children (<8 years-old). However, many studies confirmed the advantages of statin use in young FH children in a safe manner [92,94]. The youngest HoFH patients, initiating a statin treatment was a Chinese boy of only 3 years of age in 2001 [95]. Different studies support the statin benefits in young FH patients initiating the therapy in their childhood [96]. A 20-year long-term study was published [97]. It showed that FH patients who started statin treatment in childhood had a slow progression of carotid intima–media thickness and a reduced risk of CVD and death from CV causes in adulthood [97]. These studies provided important evidence about the need for an early and aggressive treatment in both forms of the disease (maybe even earlier and with lower targets than the ages and the goals recommended by current guidelines) in order to reach not only a simple reduction in LDL-C but also an improvement in coronary outcomes and survival.

## 6. LDL-C Changing Paradigm: Implication in Therapeutic Management

Following the principle of “the lower the better”, along with the notions of “the earlier the better” and “the longer the better”, contemporary lipid therapy guidelines prioritize reducing ASCVD risk, regardless of the mechanism of action of the LLT. A 54.5% reduction in the risk of CHD can be obtained by long–term exposure to lower blood LDL–C [44].

**Figure 3:**
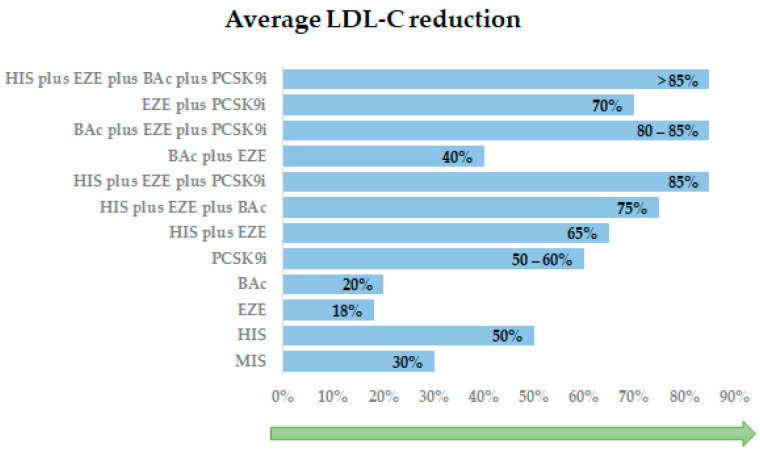
Efficacy of lipid—lowering therapy in reducing LDL. MIS: moderate-intensity statin; HIS: high-intensity statin; EZE: ezetimibe; BAc: Bempedoic Acid; PCSK9i: proprotein convertase subtilisin/kexin type 9 inhibitor.

The contribution of statins, competitive inhibitors of hydroxymethylglutaryl (HMG) CoA reductase, in reducing ASCVD risk in both primary and secondary prevention is now well-established [98]. For every mmol/L reduction in LDL-C, regardless of initial lipid profile or other initial presenting characteristics, statins can safely reduce the 5-year incidence of MACE, coronary revascularization, and stroke [99]. Moderate-intensity therapy reduces LDL-C by 30% to 50% from baseline, whereas a high-intensity regimen may achieve reductions > 50% from baseline [21]. In patients recovering from acute coronary syndrome, an intensive lipid-lowering statin regimen offers superior protection against death, or MACEs, compared to a standard regimen [100]. These results emphasize the importance of aggressively lowering LDL cholesterol levels well below current targets for improved outcomes in this patient population [100]. However, more recently, the old dogma of a high-intensity statin regimen has moved to the new concept of a high-intensity lipid-lowering strategy [7], recommending a combination therapy as the primary step for patients with a huge gap to target. In this regard, adding ezetimibe and/or bempedoic acid and/or PCSK9i is efficient in achieving an LDL-C reduction as high as 85% or more from baseline [35], as shown in Figure 3.

The monotonic relationship between lower achieved LDL-C levels—down to very low levels < 20 mg/dL—and a lower CV risk confirms that “the lower is better” [78].

Moreover, since the reduction in the risk of CV events is proportional to the extent of the reduction in LDL-C [5,44], it is easy to realize how an aggressive LLT can and should be conceived and pursued from the early stages of the treatment of dyslipidemia. This could lay the foundations for a LDL-changing paradigm in therapeutic management.

## 7. LDL-C as a Causal Risk of ASCVD: Pro and Contra

Atherosclerosis is a systemic disease resulting from a combination of lipid deposition (mainly the oxidized form), immune-inflammatory modulation, cellular proliferation and migration, extracellular matrix degradation, and thrombus formation [11]. Endothelial dysfunction and lipid accumulation within the sub-endothelial space lead to “fatty streak” formation that represents the atherosclerotic primary lesion [11]. Cholesterol accumulation is the main trigger in this process, modulating the immune-inflammatory response and lesion progression [11]. Thus, considering LDL-C only as a risk factor may be reductive.

A Consensus Statement from the EAS Consensus Panel, published in 2017, underlined that the association between LDL-C and ASCVD satisfied all causality criteria (plausibility, strength, biological gradient, temporal sequence, specificity, consistency, coherence, reduction in risk with intervention). This evidence was based on prospective epidemiological studies, Mendelian randomization studies (which studied different gene variants associated with lower LDL-C levels, randomly inherited by offspring) and randomized clinical trials (that analyzed LDL-C lowering therapies effects) [5]. The final conclusion of these studies was unique: a dose-dependent log-linear association between LDL-C levels and the ASCVD risk that increases with time exposure, with advantages derived from LDL-C reduction independently by strategies used to reduce LDL-C [5].

However, this causal link was confuted by a critical review from a Japanese group [101]. In this article, it was evidenced that in particular population groups, there was not a significant positive association between LDL-C levels and ASCVD risk [101]. According to these authors, the EAS hypothesis that LDL-C causes ASCVD is rather risky. They summarized pharmacological/biochemical studies indicating that atherosclerosis is caused by statins taken to lower LDL-C, as well as by warfarin and other vegetable fats and oils, in the absence of significantly elevated LDL-C levels. Thus, they stated that the promotion of statin treatment is not justified. Furthermore, they pointed out that a lifetime exposure to low levels of LDL-C and LDL-carried lipids, particularly fats used for energy, might be dangerous for cardiac muscle performance. For this reason, exposure to low LDL-C levels can be an ischemic risk factor, especially in older people [102]. Moreover, because LDL-C is a key component of many hormones and all cell membranes, it has been suggested that lowering LDL-C too far might interfere with cellular functions, especially in organs with higher lipid concentrations, such as the brain and reproductive organs. Additionally, a slightly increased risk of osteoporosis and brain bleeding in patients treated with high doses of statin medications has been reported [102]. However, these findings are not supported by systematic reviews and meta-analyses [103].

Another important issue to be considered is that LDL-R gene mutation (the most important alteration of FH) can involve some other genes of the same locus or close to it (as genes for the coagulation factors IX, X, protein C, tumor necrosis factor alfa, and epidermal growth factor), which could be additional determining factors of endothelial/arterial dysfunction. They finally stated that a significant correlation between LDL-C-lowering drug effects and ASCVD mortality risk reduction is not demonstrated [101].

With respect to this analysis and this point of view, several different studies have shown and replicated the major relationship between LDL-C and ASCVD [1,4,8,10,60,104,105,106]. Moreover, interventional trials have clearly indicated that LLT, not only statins, reduce LDL-C and, consequently, MACEs [6,9,20,22,23,37,60,74,78,89,107,108,109,110,111]. Certainly, there is a residual risk, non-LDL-C-related, that should be taken into account and opportunely treated [112,113,114,115].

Therefore, despite an optimized treatment to reduce LDL-C, the recurrence of CV events is based on residual CV risk, which includes some other factors such as triglyceride-rich lipoproteins (TRLs), remnant cholesterol (RC), lipoprotein(a) [Lp(a)], chronic inflammation, obesity, diabetes, and lifestyle habits. For this reason, to control non-LDL-C CV residual risk as inflammation, the use of colchicine has been suggested, as supported by the available literature [116]. Finally, new therapeutic strategies to reduce TRLs, RC (as inhibition of apolipoprotein C-III or angiopoietin-related protein-3), and Lp(a) (as anti-sense oligonucleotides and small-interfering ribonucleic acid) have been prospected [112,113].

## 8. Discussion and Conclusions

In the present article, we have summarized the available evidence on the causal role of LDL-C in the development of ASCVD. The majority of data published to date supports this causality role, which has been recognized by the current guidelines, delivering to the scientific community new important messages. First, the limit between primary and secondary CV prevention is becoming thinner and less defined [117]. Second, LCL-C should be low for primary prevention [20,60,118] and even lower for secondary prevention [21,107,119,120]. A desirable LDL-C target of at least 100 mg/dL (or even 70 mg/dL) in primary prevention should be considered because it is associated with increasing CV benefits and socio-economic impact [65]; third, the target should be achieved early since the delay seems to be associated with a gap that is not recoverable overtime [9,78]; and finally, the maintenance overtime of the suggested targets is mandatory to preserve the CV benefits [9,22,78,121,122,123]. Because of its causal role in CVD and taking into account decades of evidence accumulated in the literature, the notion of LDL-C as a risk factor free from any SCORE should be supported [124], and its early evaluation in younger people should be suggested and pursued to limit its cumulative effect in the long term, thus preventing CVD development. According to the present analysis, this should be the main message to deliver. As for any infectious diseases in which eradication of the infective agent is the only strategy available to restore health and wellness, “eradication” of LDL-C is probably the primary intervention to reduce the burden of ASCVD. Cardiovascular and cerebrovascular complications of ASCVD are a true global pandemia, and LDL-C is the etiological agent.

## Figures and Tables

**Table 1:** LDL-C targets according to 2019 ESC/EAS guidelines and 2023 Updated EAS consensus panel.

Risk Categories	LDL-C Target
FH patients at very-high risk of ASCVD	≥50% reduction in LDL-C from baseline and LDL-C < 55 mg/dL.
FH patients at high risk of ASCVD	≥50% reduction in LDL-C from baseline and LDL-C < 70 mg/dL.
FH children >10 years of age	LDL-C < 135 mg/dL *
FH children <10 years of age	≥50% reduction in LDL-C *
HoFH children <18 years of age Without ASCVD ^&^	LDL-C < 115 mg/dL *

* A very high LDL-C or additional cardiovascular risk factors may decrease this target. ^&^ A lower goal is recommended for those with established ASCVD.

**Table 2:** When to start medical treatment in FH children?

Guidelines	Age of Beginning of Statins
2014 EAS consensus panel	8–10 years of age
2019 AHA/ACC guidelines	10 years of age
2019 ESC/EAS guidelines	6–10 years of age *
2023 Updated EAS consensus panel	at diagnosis in children with HoFH

* A very high LDL-C or additional CV risk factors may decrease the age recommended for starting statin therapy.

## Data Availability

The data from this manuscript are derived from publicly available published clinical trial and study results.
